# Molecular Characterization of Colistin Resistance *Acinetobacter baumannii* Isolates and Identification of Mutations in *pmrA* and *lpxA* Genes from a Tertiary Care Hospital in South India

**DOI:** 10.3390/antibiotics15090846

**Published:** 2026-08-31

**Authors:** MuthuLakshmi BackiaSubramanian, Madhumala Shanmugasundaram, Shanthi Mariappan, Uma Sekar, Renuka M K, Thyagarajan Ravinder

**Affiliations:** 1 Department of Microbiology, Sri Ramachandra Institute of Higher Education and Research, Chennai 600 116, Tamil Nadu, India; muthulakshmib@sriramachandra.edu.in (M.B.); madhumalas@sriramachandra.edu.in (M.S.); umasekarsrmc@gmail.com (U.S.); 2 Department of Critical Care Medicine, Sri Ramachandra Institute of Higher Education and Research, Chennai 600 116, Tamil Nadu, India; renuka.mk@sriramachandra.edu.in; 3 Department of Microbiology, Government Kilpauk Medical College, Chennai 600 010, Tamil Nadu, India; drtravinder67@gmail.com

**Keywords:** colistin resistance, *Acinetobacter baumannii*, *pmrAB*, *lpxACD*, healthcare

## Abstract

**Background/Objectives**: *Acinetobacter baumannii* is a multidrug-resistant (MDR) nosocomial pathogen associated with significant morbidity and mortality. The emergence of strains resistant to multiple antibiotics has markedly reduced available treatment options. *A. baumannii* now demonstrates resistance to most first-line antibiotics, resulting in the extensive use of colistin, which in turn has led to the emergence of colistin-resistant strains. There are very limited studies from India on the mechanisms of colistin resistance. In this study, we focused on the molecular mechanisms leading to colistin resistance. **Methods**: A total of 225 clinical isolates of *Acinetobacter baumannii* were analyzed in this study. Antimicrobial susceptibility testing was done using the disk diffusion method for various classes of antimicrobial agents as per the Clinical Laboratory Standards Institute (CLSI M100 Ed 35,2025). Susceptibility to colistin was tested by the Microbroth dilution method. Colistin resistance mediated by the *mcr* gene and alterations in the *pmrAB* and *lpxACD* genes were investigated using conventional PCR followed by mutational analysis. **Results:** Among the 225 isolates, 4 isolates were found to be colistin resistant. All the resistant isolates had an MIC ≥ 16 mcg/mL. Mutations were found in *lpxA* and *pmrB* (c.391T > C, c.495T > C, c.516A > G, c.732A > G and c.966T > C). Mutations in *pmrA*, *lpxC*, and *lpxD* were not detected in this study. Mcr-1 gene was not detected in this study. **Conclusions**: Colistin is the last resort for the treatment of MDR strains, and the increase in resistance to colistin has become a huge concern among the medical community as it reduces the options available for treatment. This is the first report on the molecular mechanisms of colistin resistance in South India using PCR. The study is also the first to report four novel mutations in the genes that may be responsible for resistance. Early detection of resistance and swift control measures can help curb the morbidity and mortality rate among such *A. baumannii* strains.

## 1. Introduction

*Acinetobacter baumannii* is a Gram-negative opportunistic pathogen causing nosocomial infections. It is named as one of the priority pathogens for research by the WHO [1]. The priority status is attributed to the organism’s remarkable capacity to gain antimicrobial resistance through various intrinsic and acquired mechanisms [2,3]. The common choice of treatment for MDR Gram-negative organisms is usually Carbapenems [4,5]. However, rapid emergence of carbapenemase producing *A. baumannii* has been observed in clinical settings. It has also been observed that there has been a swift increase in strains that are resistant to multiple antibiotics, including fluoroquinolones, tetracyclines and aminoglycosides [6]. This has significantly reduced the antibiotics available, eventually leading to colistin being the only remaining antibiotic for treatment of infections caused by MDR *A. baumannii*.

Colistin (Polymyxin E), a member of the polymyxin class of antibiotics was first discovered in 1947 as secondary metabolites of *Paenibacillus polymyxa* [7]. It is a bactericidal cyclic decapeptide that attaches to the negatively charged outer membrane surface of the organism, resulting in disruption of the cellular membrane and eventually cell death [8]. Colistin has a D-leucine in its structure and is biologically similar to polymyxin B. Both have been frequently used in clinical settings; however, their use were restricted in 1970 due to neurotoxicity and nephrotoxiity after prolonged use [9].

Unfortunately, carbapenem resistance in *A. baumannii* has led to the overuse of colistin, giving rise to colistin-resistant isolates [10]. This overuse often gives rise to resistant strains due to the increase in selective pressure [11].

*Acinetobacter baumannii* employs multiple mechanisms to overcome the antibacterial activity of polymyxins. These include modification of the outer membrane through the addition of phosphoethanolamine to lipid A, loss of lipopolysaccharide, outer membrane asymmetry disruption, metabolic adaptations involving osmoprotective amino acids, and overexpression of efflux pumps [12]. Based on origin, the mechanism of resistance is classified as chromosomal or plasmid-mediated. It should be noted that the main mechanism for resistance is of chromosomal origin, leading to changes in the outer membrane.

The first and most common mechanism involves the total loss of lipid A, resulting in a lipopolysaccharide-devoid outer membrane, caused by spontaneous point mutations in one of the genes responsible for lipid A biosynthesis (*lpxA*, *lpxC*, *lpxD*) [13]. The second mechanism of resistance is regulated by the pmrCAB operon; it alters the lipid A component of the LPS. The two-component *PmrA/B* governs *PmrC* expression, which facilitates the addition of phosphoethanolamine (pEtN) to lipid A. The outer membrane negative charge is lowered, reducing the binding of colistin to the cell membrane [14]. The third mechanism involves mutations in the emrB gene family leading to the activation of broad-spectrum efflux pumps or causing change in membrane permeability by ompA porin entry channels [15].

The plasmid encoded mcr-1 gene plays an important role colistin resistance. This gene was first identified in *Escherichia coli* in China in 2015 [16]. Since then, variants of this gene (mcr 2-5) have been identified in various parts of the world [17,18,19]. Phosphoethanolamine transferase is encoded by the mcr gene [4]. It facilitates the addition of phosphoethanolamine generated from the cleavage of phosphotidylethanolamine, eventually leading to changes in the LPS layer [20,21]. The advent of plasmid-mediated genes in polymyxin resistance isolates that can spread easily from one bacterium to another is a matter of significant concern.

Polymyxin-resistant *A. baumannii* currently make up a mere one percent of clinical isolates, but they present an urgent concern in health care settings [22]. Globally, there have been various studies on the prevalence of colistin resistance in *A. baumannii*, but when the focus shifts to molecular mechanisms, only minimal literature is available, especially in the Indian subcontinent, masking the true burden of the emerging problem. In light of these limitations, this study aims to address the gap by detecting and characterizing the mutations in selected genes previously associated with colistin resistance in *A. baumannii* isolates.

## 2. Results

### 2.1. Specimen Distribution

A total of 225 *A. baumannii* isolates, were obtained from various clinical specimens. The majority of the isolates were collected from respiratory samples (79), followed by exudates (77), blood (52) and urine (17).

### 2.2. Antimicrobial Susceptibility Test

The Kirby–Bauer method was used for antimicrobial susceptibility testing for all 225 isolates, of which the MIC pattern of the colistin resistance strains is given in Table 1. Four isolates were found to be colistin resistant (ColR), and their respective MIC ranged from 16 mcg/mL to ≥64 mcg/mL using the Microbroth dilution method. Furthermore, the MIC range of all 225 isolates is shown in Table 2

### 2.3. Patient Demographic Details

All of the patients harboring colistin-resistant strains were admitted to the ICU. Prior administration of colistin was not noted in any of the patients before the resistance was identified. However, prior exposure to carbapenems was seen in all isolates. Detailed patient information is provided in Table 3.

### 2.4. Study of Colistin Resistance Mechanisms

**Figure 1:**
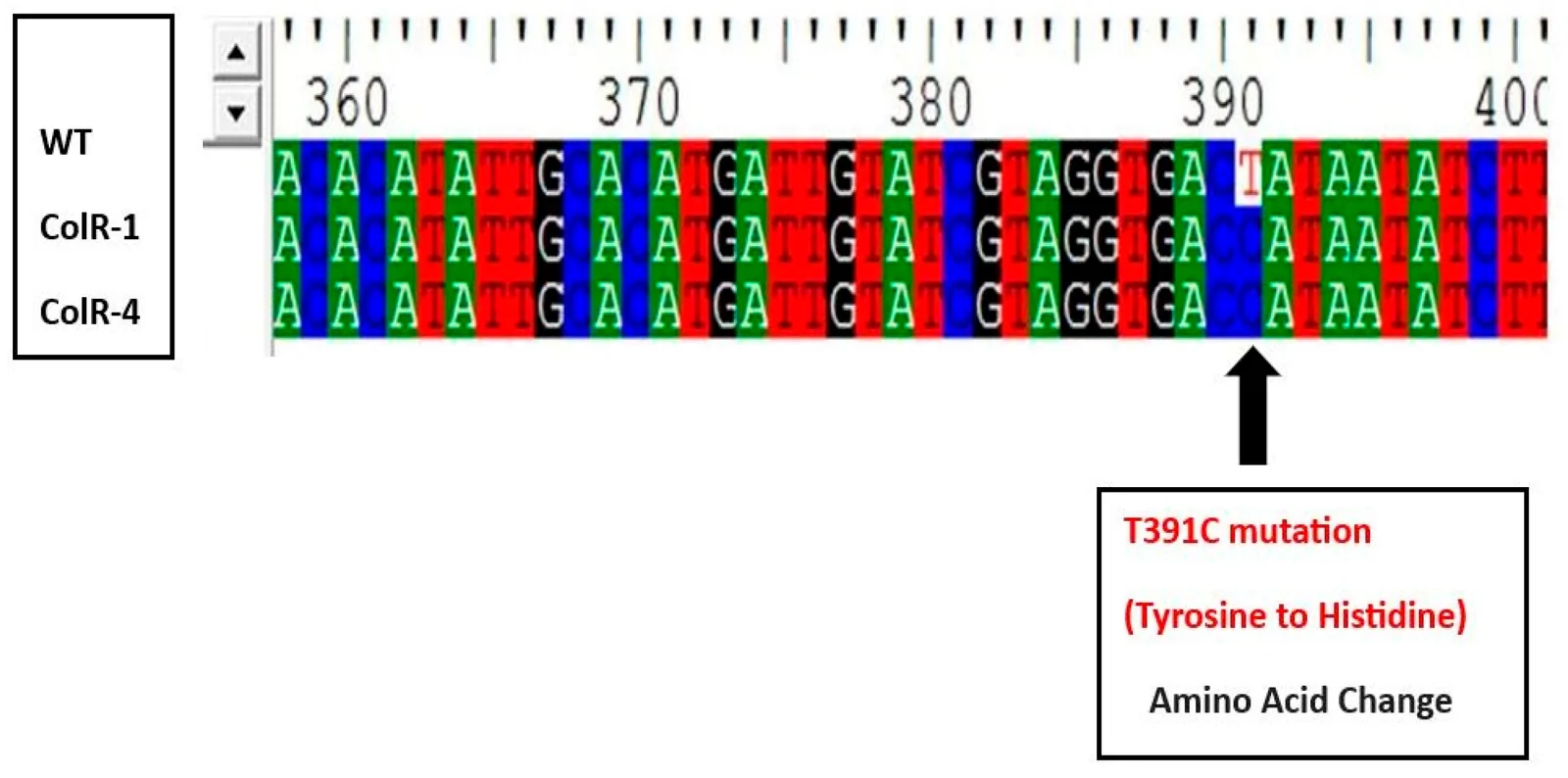
Representative image of multiple sequence alignment of T391C mutation in the lpxA gene of clinical isolates (ColR-1 and ColR-4 compared to the Wild type (WT).

The colistin-resistant isolates (*n* = 4) had an MIC of ≥16 mcg/mL. All four isolates were screened for the presence of mutations in the *lpxACD* and *PmrAB* genes. Five distinct point mutations were observed in two of the four col-R isolates as tabulated in Table 4. Full length sequencing of two isolates of the *lpxA* gene identified a non-synonymous mutation (c.391T > C/p.Tyr131His) leading to an amino acid substitution (Tyrosine to Histidine), as shown in Figure 1; the same isolates also harbored three synonymous mutations. Both the isolates demonstrated elevated MIC of 16 mcg/mL and 64 mcg/mL. All four were substitution mutations in the *lpxA* gene. No mutations were reported in the *lpxC* and *lpxD* genes.

Both *pmrA* and *pmrB* genes were detected. Of these, no mutations were recorded in pmrA. One substitution mutation was detected in *pmrB* after the SNP analysis. However, no amino acid change was detected. The mcr gene responsible for horizontal gene transfer was not identified in this study. Among the 4 Col-R isolates, mutations were found only in ColR-1 and ColR-4; the mechanism of resistance could not be decoded for ColR-2 and ColR-3. Coexistence of *pmrB* and *lpxA* was noted in ColR-4.

**Figure 2:**
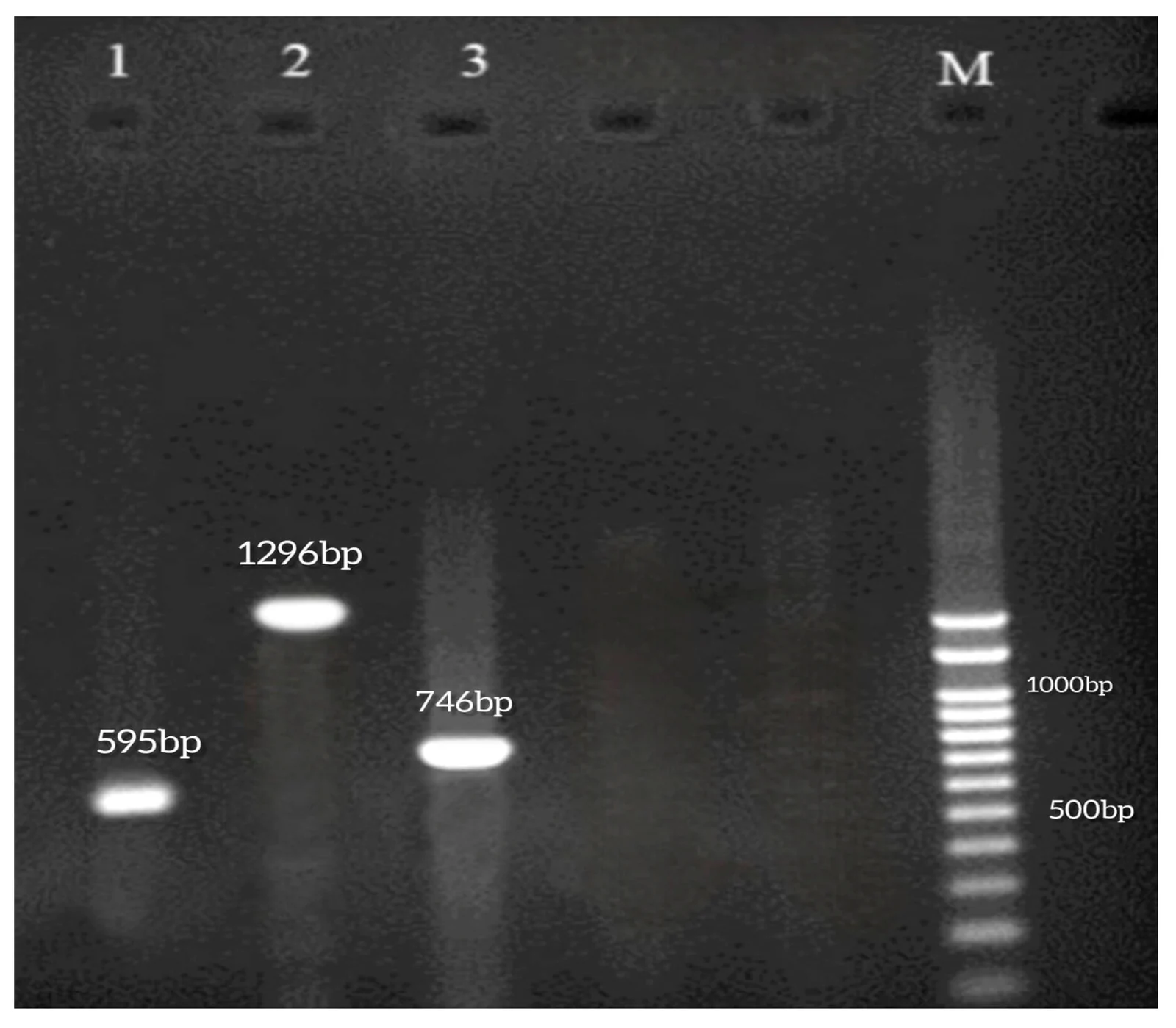
Agarose gel electrophoresis of PCR products corresponding to colistin-resistant genes *pmrA*, *pmrB*, and *lpxA*. Lane 1—*pmrA* (595 bp); Lane 2—*pmrB* (1296 bp); Lane 3—*lpxA* (746 bp); M—DNA Marker.

Representative amplicons were deposited in GenBank under the accession numbers (*pmrA* -PZ315971, *pmrB*- PZ315972, and *lpxA* -PZ315970 were obtained. The representative gel electrophoresis image is depicted in Figure 2.

### 2.5. Statistical Analysis

Statistical analysis was performed for colistin-resistant and colistin intermediate isolates of *Acinetobacter baumannii.* Among the 225 total isolates, four were resistant to colistin, corresponding to a prevalence of 1.78% (95% exact confidence interval: 0.49–4.49%). All colistin-resistant isolates were recovered from ICU patients; however, no statistically significant association was observed between ICU admission and colistin resistance. Fisher’s exact test was performed due to the small sample size and low expected frequencies in the comparison group (Table 5).

## 3. Discussion

*Acinetobacter baumannii* is a multidrug-resistant (MDR) nosocomial pathogen associated with severe healthcare-associated infections. The emergence of multiple antibiotic-resistant strains has significantly reduced available treatment options and increased morbidity and mortality. Resistance to most first-line antibiotics has resulted in the extensive use of colistin as a last-resort therapy against Gram-negative bacterial infections. However, the widespread use of colistin has subsequently led to the emergence of colistin-resistant strains [23]. Despite this growing concern, studies exploring the molecular mechanisms of colistin resistance in India remain limited. In the present study, we investigated the molecular mechanisms associated with colistin resistance in *A. baumannii* isolates.

The prevalence of colistin resistance among isolates in this study was 1.78%, which is comparable to resistance rates reported in European studies ranging from 0 to 4% [24]. Our findings are also broadly comparable to those reported by Sharma et al., 2.59%, in 2020 [1]. Although both studies included clinical isolates from a variety of specimen types and employed PCR-based molecular analysis, they were conducted in different geographic regions of India; the slight variation in colistin resistance rates may also be due to differences in circulating strains or patient practices. Therefore, direct comparisons between these studies should be interpreted with caution. In contrast, significantly higher resistance rates have been documented globally, including Spain (19.1%), Greece (27%), and Bulgaria (6.7%), highlighting the growing international concern regarding colistin resistance in *A. baumannii* [25,26,27,28]. Data from India, however, remain limited, with only a few notable reports by Sharma et al. (2020) and Vijayakumar et al. (2024) [1,29].

The present study demonstrated that colistin-resistant (Col-R) isolates also exhibited resistance to several other antibiotics, including amikacin, piperacillin–tazobactam, ciprofloxacin, ceftazidime, imipenem, and meropenem. Universal susceptibility was observed for tigecycline. All Col-R isolates were recovered from patients admitted to intensive care units, emphasizing the highly resilient nosocomial nature of *A. baumannii* [15].

Two isolates, ColR-1and ColR-2, were isolated from respiratory samples with MICs of 16 mcg/mL and 32 mcg/mL, respectively. ColR-3 and ColR-4 were isolated from exudative specimens and showed higher MICs of ≥64 and 64 mcg/mL. This difference in MIC between respiratory and exudative isolates may be because of the increased biofilm-forming capacity of *A. baumannii* isolates associated with soft tissue infections, which may severely restrict antibiotic penetration and promote adaptive resistance [30]. Furthermore, the chronic nature of wound infections is often linked to frequent exposure to antibiotics. However, this requires further investigation.

In this study, no prior colistin exposure was documented among patients with colistin-resistant isolates. Although previous colistin exposure is considered a major risk factor for the development of resistance [31] its absence in the present study suggests the involvement of alternative resistance mechanisms. Nevertheless, colistin resistance is generally driven by selective pressure leading to mutations in specific genetic determinants. All patients had at least one indwelling device, and three of the four patients had significant comorbidities, both of which are established risk factors that may have contributed to the acquisition and persistence of *A. baumannii* infections.

Globally, colistin resistance has been increasingly reported since 2010 [13]. In South India, a similar trend was observed around 2017, when early clinical studies began documenting the emergence of colistin-resistant *A. baumannii* isolates [32]. According to the previous literature, the mechanisms of colistin resistance are believed to be primarily associated with alterations in the *pmrAB* and *lpxACD* genes. In the present study, four isolates were confirmed to be colistin resistant. Notably, all isolates harbored the *pmrAB* and *lpxA* genes, and SNPanalysis using CLUSTAL W 2.1 software identified one mutation in the *pmrB* and four mutations in the *lpxA* genes, respectively.

Variants in the *pmrB* gene, including frameshifts, deletions, and non-synonymous SNPs, have previously been associated with colistin resistance [13]. In the present study, to the best of our knowledge, a previously unreported synonymous substitution in *pmrB* (c.966T > C/Ile) was identified. Other *pmrB* mutations reported in the literature include A138T [27] and two T-deletion mutations at nucleotide positions 225 and 232, described by Sharma et al. from India [1]. Globally, several amino acid substitutions have been documented. Farjania et al. (2022) reported mutations such as V162A, A371V, and V133D [10], whereas Beceiro et al. and Park et al. described multiple mutations including I121F, T192I, Q228P, A184V, P190S, A183T, L87F, M145K, S14L, P233S, A227V, S403F, F387Y, and N353Y [33,34]. A wide spectrum of mutations has also been reported from Spain, Israel, Brazil, and the USA [35]. Interestingly, none of these previously reported mutations were identified in the present study. However, several studies have reported at least one point mutation in all colistin-resistant isolates, indicating that *pmrB* is more prone to mutations than *pmrA* [10,15].

No mutations in *pmrA* were detected in this study, consistent with findings from Iran [10]. Previous studies have shown that *pmrA* mutations are infrequently associated with colistin resistance [14]. Nevertheless, sporadic mutations have been reported, including the M12I substitution described by Vijayakumar et al. and the A76T and D14N substitutions reported by Sharma et al. from India [1,29]. Additional mutations reported globally include S119T from Spain [35], G54E from Saudi Arabia [36], and P102H from Malaysia [37].

Mutations in *lpxACD* are regarded as major mechanisms underlying colistin resistance, primarily through loss of the lipopolysaccharide layer, which contributes to high-level resistance. In the present study, two isolates demonstrated identical mutations (c.391T > C/p.Tyr131His, c.495T > C/Asp, c.516A > G/Gly, and c.732A > G/Pro) and exhibited elevated MICs of 16 mcg/mL and 64 mcg/mL. The highest number of mutations was observed in the *lpxA* gene. The non-synonymous mutation p.Tyr131His was previously reported and can be considered a candidate resistance associated variant, although functional validation is required to confirm its role in colistin resistance [29]. This finding is consistent with reports from Iran, where all colistin-resistant isolates harbored mutations in *lpxA* genes, including 299V→G, 292Y→H, 394E→D, 342P→R, 405P→R, 107K→Q, 283I→N, and frameshift mutations, highlighting their central role in resistance [10,15].

In the Indian context, data regarding *lpxA* mutations remain scarce. Sharma et al. reported two deletion mutations (at nucleotide positions 44 and 594) and one insertion mutation (at nucleotide position 621) [1], whereas Vijayakumar et al. identified the Y131H or c.391T > C/p.Tyr131His substitution which was detected in this study [29]. Importantly, four synonymous mutations identified in the present study appear to be novel. While synonymous mutations do not alter protein sequence, numerous studies in bacteria show they can markedly change mRNA behavior and protein yield. Silent SNPs have been shown to up- or down-regulate expression of resistance enzymes and fitness, via effects on mRNA structure, translation initiation, and stability. Therefore, resistance-associated genes harboring novel silent variants should be interpreted with caution, such variants may contribute to the resistance phenotype even though the protein sequence is unchanged [38]. Thus, the contribution of these synonymous variants to colistin resistance remains uncertain and requires functional validation.

No mutations were identified in the lipid A biosynthesis genes *lpxC* and *lpxD*. This observation is in agreement with several recent studies reporting colistin-resistant isolates lacking mutations in these genes. Ahsan et al. (2022) described a colistin-resistant strain possessing intact *lpxC* and *lpxD* genes [39]. Similarly, a Korean surveillance study failed to detect mutations in these genes. An Indian hospital based study identified nine novel mutations but explicitly reported no mutations in *lpxC* [1]. However, a few studies have identified mutations in these genes as well [10,29].

The diversity of mutations reported across studies suggests that the molecular mechanisms underlying colistin resistance in *Acinetobacter baumannii* may vary geographically. Differences in antibiotic usage patterns, local selective pressures, and clonal distribution may contribute to the observed regional variation in resistance-associated mutations [1,10,29].

In addition to chromosomal mechanisms, plasmid-mediated resistance through the *mcr-1* gene was also screened in this study. None of the isolates harbored the *mcr-1* gene. Consistent with previous reports, *mcr*-mediated resistance in *A. baumannii* remains rare, with the first reports from India and Pakistan published only in 2019 [40,41].

The resistance mechanisms in two of the four colistin-resistant isolates could not be identified, one of which demonstrated the highest colistin MIC observed in this study. This may be due to alternative mechanisms involving genes such as *lpsB*, *lptD*, and *vacJ*. The *lpsB* gene contributes to LPS core biosynthesis, while *lptD* is involved in LPS insertion into the outer membrane. The *VacJ* (*Vps*) ABC transporter system is believed to maintain lipid asymmetry, which is critical for membrane integrity. Additionally, efflux pumps, particularly those belonging to the major facilitator (MF) family such as EmrAB, have also been implicated in multidrug resistance [12]. Collectively, these alternative mechanisms may explain the unexplained resistance observed in two isolates in this study. Future studies employing whole-genome sequencing and functional analyses may provide a more comprehensive understanding of the resistance mechanisms in these isolates.

This single-center study provides insight into the molecular epidemiology of colistin-resistant *A. baumannii* in our institution. However, only four colistin-resistant isolates were identified, limiting statistical analysis and the generalizability of the findings. Furthermore, only selected resistance genes were investigated, and susceptible isolates were not sequenced for comparison. Therefore, it was not possible to determine whether the identified mutations were unique to resistant isolates or represented naturally occurring polymorphisms. Further comparative sequencing is required to establish their role in colistin resistance. Functional validation was not performed; therefore, the contribution of the identified mutations, particularly the synonymous substitutions, to colistin resistance remains uncertain. Nevertheless, this study expands the available molecular data on colistin resistance from India and provides a foundation for future genomic and functional investigations.

## 4. Materials and Methods

### 4.1. Study Site and Ethics Approval

This in vitro laboratory-based prospective study was conducted in the Department of Microbiology of a 1600 bedded university teaching hospital. The study was approved by the Institutional Ethics Committee (IEC-NI/23/AUG/88/42) dated 25 August 2023.

### 4.2. Bacterial Strain

The study included a total of 225 consecutive, non-duplicate, clinically significant *A. baumannii* isolates, screened from August 2023 to August 2024. The isolates were obtained from various clinical samples such as blood, lower respiratory tract secretions (endotracheal secretions, bronchoalveolar lavage and bronchial wash), wound exudates, pus and urine. The study excluded isolates from outpatients, repeated isolates from the same patient, and isolates suspected to be colonizers. Both the traditional and automated methods—VITEK^®^MS MALDI-TOF (bioMerieux, Marcy l’Etoile, France)—were utilized to identify the organism up to species level. The isolates were considered clinically significant based on relevant clinical history, demonstration of organisms on Gram stain, presence of intracellular forms, and significant pure culture growth.

### 4.3. Antimicrobial Susceptibility Testing

Kirby–Bauer method was used to determine the antimicrobial susceptibility of various classes of antibiotics. The antibiotics tested were amikacin (30 mcg), ciprofloxacin (5 mcg), ceftazidime (30 mcg), piperacillin tazobactam (100/10 mcg), imipenem (10 mcg) and meropenem (10 mcg), (Himedia Laboratories Mumbai, Thane, Maharashtra, India). Antimicrobial susceptibility results were interpreted according to the Clinical and Laboratory Standards Institute (CLSI) M100, 35th Edition (2025).

### 4.4. Minimum Inhibitory Concentration

Estimation of MIC for colistin and tigecycline was performed using Microbroth dilution method, using Micropro BM kit (Microexpress, Verna, Goa, India). The kit includes Broth microdilution strips with wells coated in 2-fold dilutions of the antimicrobial agent (colistin). The range of the pre-coated antibiotic in each well is from 0.06 mcg/mL to 64 mcg/mL for colistin and 0.03 mcg/mL to 32 mcg/mL for tigecycline. 200 µL of the bacterial inoculum broth is then added to the wells, and they are incubated at 35 °C overnight. According to the 2025 CLSI guidelines, the MIC is calculated by determining the lowest concentration of colistin that visibly inhibits the bacterial growth. Strains that have an MIC of ≤2 mcg/mL is considered as intermediate and MIC of ≥4 mcg/mL is considered resistant [1]. For tigecycline, the MIC was interpreted according to the most recent Food and Drug Administration guidelines 2023 for enterobacterales with strains that have an MIC of ≤2 mg/L considered sensitive, 4mg/L considered intermediate and ≥8mg/L considered resistant [42].

### 4.5. Molecular Methods

#### 4.5.1. DNA Extraction

Extracted DNA from the isolates were obtained by using the boiling lysis procedure. Single colony from an overnight culture is inoculated in 400 µL TE buffer or Double autoclaved distilled water. This suspension was subjected to a 10 min boiling process at 95 °C, followed immediately by a 10 min freezing period at −20 °C, then a 10 min centrifugation at 12,000 rpm. After retrieving the suspension that contains the DNA, the pellet was discarded [42]. For further analysis, this suspension was stored at −20 °C. A total of 2 µL of this suspension was used as template for amplification in Polymerase Chain Reaction.

#### 4.5.2. Polymerase Chain Reaction

PCR was done to identify genes that were responsible for complete loss of LPS layer (*lpxA*, *lpxC*, *lpxD*) and for the two component system genes (*pmrA*, *pmrB*). All the positive samples were checked for detection of mutations. The primers used for characterization are given in Table 6. PCR reaction for *lpxA*, *lpxD* and *pmrA* began with an initial denaturation at 94 °C for 4 min, 40 cycles of (94 °C for 1 min, 49 °C for 1 min, and 72 °C for 1 min); the final extension was carried out at 72 °C for 5 min. The annealing temperature for *lpxC* was 52 °C for 1 min; all the other conditions were the same as above. For *pmrB* conditions, initial denaturation at 94 °C for 3 min, 94 °C for 30 s, 30 cycles of 55 °C for 30 s, 72 °C for 45 s and final extension at 72 °C for 5 min were used.

The final volume of all PCR reaction was 25 µL. A total of 10 pmol of each primer was added with 23 µL of high fidelity mastermix (Immugenix Biosciences, Chennai, India) and 2 µL of template DNA was added to each reaction.

Reactions for mcr 1 gene were carried out using the following conditions: initial denaturation at 94 °C for 3 min, denaturation (25 cycles) at 94 °C for 30 s annealing at 52 °C for 30 s, 72 °C for 1 min, and final extension cycle at 72 °C for 10 min. The final volume of the mcr-1 gene PCR reaction was 10 µL, with 5 µL mastermix (Applied Biosystem, Foster City, CA, USA), 2 µL miliQ water, 0.5 µL of each primer and 2 µL of template DNA.

The amplicons were separated using 2% agarose gel (Bioworld, Banglore, India) with Ethidium Bromide. Strains previously characterized using DNA sequencing were used as positive controls and sterile Mili Q water was used as negative control.

#### 4.5.3. DNA Sequencing and Analysis

The positive amplicons from the PCR reactions were purified using FavorPrep PCR Purification Mini Kit (Favorgen, Tapei City, Taiwan) and were subjected to Sanger sequencing using ABI 3500XL sequencer (Applied Biosystems, Foster City, CA, USA) with using ABI PRISM BigDye Terminator kit Sequences were aligned using BioEdit software version 7.0.5.3 and similarity searches were performed using the Basic Local Alignment Search Tool (BLAST) version +2.17.0 available through the National Center for Biotechnology Information (NCBI) database 

The DNA sequence obtained was submitted to gen bank and accession numbers were obtained. For the mutation analysis, the Clustal W Multiple sequence alignment tool version 2.1 was used and any mutations in the nucleotide sequence were noted.

Clinical, demographic and relevant treatment-related information of patients from whom Col-R strains were isolated were collected retrospectively from the medical records department.

#### 4.5.4. Statistical Analysis

All statistical analysis was performed using Graph pad prism version 11.0.2. The prevalence of colistin resistance was calculated as the proportion of resistant isolates among the total number of isolates studied and expressed with a 95% exact binomial confidence interval (Clopper–Pearson method). Categorical variables were compared using Fisher’s exact test owing to the limited number of colistin-resistant isolates. A two-tailed *p*-value of <0.05 was considered statistically significant.

Due to the low prevalence of colistin resistance and the limited number of resistant isolates identified, advanced inferential analyses such as multivariable logistic regression were not performed.

## 5. Conclusions

Colistin continues to serve as a last-resort treatment for infections caused by carbapenemase-producing multidrug-resistant *Acinetobacter baumannii*. Nevertheless, the increasing global incidence of colistin- and carbapenem-resistant strains poses a serious public health challenge. To the best of our knowledge, this is the first study from South India to investigate the molecular mechanisms of colistin resistance using PCR and to report four novel mutations in the *pmrB* and *lpxA* genes that may contribute to resistance. These findings enhance our understanding of the genetic basis of colistin resistance and underscore the importance of early detection, molecular surveillance, and prompt infection control measures to help limit the spread of colistin-resistant *A. baumannii*. Future research should focus on functional validation of the identified mutations to determine their contribution to colistin resistance. Comparative sequencing of colistin-resistant and colistin-intermediate isolates, together with complementary molecular studies, will be essential to establish their biological significance.

## Figures and Tables

**Table 1:** AST pattern and MIC of colistin-resistant isolates.

Isolate	Disk Diffusion						Minimum Inhibitory Concentration	
	Ak	cip	caz	Pit	imp	mem	Colistin	
ColR-1	R	R	R	R	R	R	R	16 mcg/mL
ColR-2	R	R	R	R	R	R	R	32 mcg/mL
ColR-3	R	R	R	R	R	R	R	≥64 mcg/mL
ColR-4	R	R	R	R	R	R	R	64 mcg/mL

**Footnote**: Ak—Amikacin, Cip—Ciprofloxacin, Caz—ceftriaxzone, Pit—Piperacillin-Tazobactum, imp—Imipenem, Mem—Meropenem, colistin-resistant isolates-ColR-1, ColR-2, ColR-3, ColR-4.

**Table 2:** Minimum inhibitory concentration of colistin against Acinetobacter baumannii isolates.

MIC mcg/mL	0.06	0.12	0.25	0.5	1	2	4	8	16	32	64	≥64
No of isolates	0	81	58	76	6	0	0	0	1	1	1	1

**Footnote**: [Results interpreted as per CLSI guidelines 2025]. ≤2 mcg/mL—intermediate and ≥4 mcg/mL—resistant.

**Table 3:** Demographic details and clinical profile of patients with colistin-resistant Acinetobacter baumannii.

Characteristics	ColR-1	ColR-2	ColR-3	ColR-4
Age/Sex	82/Male	63/Female	59/Male	47/Male
Hospital location	ICU	ICU	ICU	ICU
MIC to colistin	16 mcg/mL	32 mcg/mL	≥64 mcg/mL	64 mcg/mL
Antimicrobial susceptibility profile	Amikacin, Ciprofloxacin, ceftriaxzone, Piperacillin-Tazobactum, Imipenem, Meropenem and Colistin	Amikacin, Ciprofloxacin, ceftriaxzone, Piperacillin-Tazobactum, Imipenem, Meropenem and Colistin	Amikacin, Ciprofloxacin, ceftriaxzone, Piperacillin-Tazobactum, Imipenem, Meropenem and Colistin	Amikacin, Ciprofloxacin, ceftriaxzone, Piperacillin-Tazobactum, Imipenem, Meropenem and Colistin
Admitting speciality	Pulmonology	Nephrology	General medicine	General surgery
Date of isolation	9 April 2023	1 April 2023	7 August 2024	14 July 2023
Specimen source	Sputum	Endotracheal aspirate	Pus	Pus
Days in the hospital	15 days	12 days	6 days	16 days
Co-morbid conditions	Type II diabetes mellitus, Systemic Hypertension	Type II diabetes mellitus	Type II diabetes mellitus, Systemic Hypertension	Nil
Underlying disease/diagnosis	Aspiration Pneumonia	Sepsis	Acute cerebrovascular accident and Urinary tract infection	Wound sepsis
Indwelling device	Peripheral line, Foley catheter, Ryles tube, endotracheal tube	Peripheral line, Ryles tube, endotracheal tube Central line	Peripheral line, Central line, Foley catheter	Peripheral line, Arterial line, Umbilical line, Foley catheter, Epidural catheter, endotracheal tube
Surgical procedure	Coronary angiogram, intubation	Intubation	Nil	Ileostomy
Prior antibiotic exposure	Piperacillin-tazobactam, Amoxicillin	Piperacillin-tazobactam, Meropenem	Piperacillin-tazobactam	Nil
Mortality	Recovered	Expired	Discharged against medical advice	Recovered

**Table 4:** Distribution of mutations among the colistin-resistant isolates of *Acinetobacter baumannii.*

Gene	Nucleotide Change	Codon Change	Amino Acid Change	Mutation Type	Synonymous/Non Synonymous	Isolate
*lpxA*	c.391T > C	TAT > CAT	p.Tyr131His	Transition	Non synonymous	ColR-1 and ColR-4
	c.495T > C	GAT > GAC	Asp	Transition	Synonymous	
	c.516A > G	GGA > GGG	Gly	Transition	Synonymous	
	c.732A > G	CCA > CCG	Pro	Transition	Synonymous	
*pmrB*	c.966T > C	ATT > ATC	Ile	Transition	Synonymous	ColR-4

**Table 5:** Contingency table showing association between ICU admissions and colistin resistance in clinical isolates of *Acinetobacter baumannii* (Fisher’s exact test).

ICU Status	Colistin Resistant	Colistin Intermediate	Total	*p* Value
ICU	4	187	191	1.000
Non-ICU	0	34	31	
Total	4	221	225	

**Table 6:** Primers and PCR conditions used for resistance genes.

Gene	Primers (5’ to 3’)	Amplicon (bp)	References
*pmr A*	FP-GGTGGAATGGGTCAATAAC RP-TTATGATTGCCCCAAACG	595	[10]
*pmr B*	FP-GAAAGAACAGCTGAGCAC RP-AACTTATGGACAGGCTGG	1296	
*lpx A*	FP-CCATTCTACCGCCATTATTGA RP-CACAATTCCACGCTCTGA	746	
*lpxC*	FP-CGTACTCTCAATCGTGTG RP-CGTATGGAATTGGACAGTC	870	
*lpxD*	FP-AAGGTGAGCTAATTGGTGAAG RP-AGTGATTTGGGTCAATGGC	959	
mcr 1	FP-CGGTCAGTCCGTTTGTTC RP-CTTGGTCGGTCTGTAGGG	309	[40]

## Data Availability

The data presented in this study are openly available in NCBI at https://www.ncbi.nlm.nih.gov/nuccore/ PZ315971 (accessed on 22 July 2026) reference number PZ315972, PZ315970.

## Abbreviations

The following abbreviations are used in this manuscript:

CLSI	Clinical laboratory standards institute
MIC	Minimum Inhibitory Concentration
PCR	Polymerase Chain Reaction
WHO	World Health Organization
MDR	Multi Drug Resistant
LPS	Lipopolysaccharide
ColR	Colistin resistant
AST	Antimicrobial susceptibility testing
ICU	Intensive Care Unit
DNA	Deoxyribonucleotide
rpm	Revolutions per minute
SNP	Single Nucleotide Polymorphism
